# The Impact of Hemolysis on Cell-Free microRNA Biomarkers

**DOI:** 10.3389/fgene.2013.00094

**Published:** 2013-05-24

**Authors:** Michaela B. Kirschner, J.James B. Edelman, Steven C-H. Kao, Michael P. Vallely, Nico van Zandwijk, Glen Reid

**Affiliations:** ^1^Asbestos Diseases Research Institute, University of Sydney, Sydney, NSW, Australia; ^2^Cardiothoracic Surgical Unit, Royal Prince Alfred Hospital, Sydney, NSW, Australia; ^3^The Baird Institute, University of Sydney, Sydney, NSW, Australia

**Keywords:** cell-free microRNA, red blood cells, hemolysis, biomarker, quality control

## Abstract

Cell-free microRNAs in plasma and serum have become a promising source of biomarkers for various diseases. Despite rapid progress in this field, there remains a lack of consensus regarding optimal quantification methods, reference genes, and quality control of samples. Recent studies have shown that hemolysis occurring during blood collection has substantial impact on the microRNA content in plasma/serum. To date, the impact of hemolysis has only been investigated for a limited number of microRNAs, mainly the red blood cell (RBC)-enriched miRs-16 and -451. In contrast, the effect of hemolysis on other microRNAs – in particular those proposed as biomarkers – has not been addressed. In this study we profiled the microRNA content of hemolyzed and non-hemolyzed plasma as well as RBCs to obtain a profile of microRNAs in the circulation affected or unaffected by hemolysis. Profiling by TaqMan Array Microfluidic Cards was used to compare three pairs of hemolyzed and non-hemolyzed plasma (with varying degrees of hemolysis) and one RBC sample. A total of 136 microRNAs were detectable in at least two of the samples, and of those 15 were at least twofold elevated in all three hemolyzed samples. This number increased to 88 microRNAs for the sample with the highest level of hemolysis, with all of these also detected in the RBC profile. Thus these microRNAs represent a large proportion of detectable microRNAs and those most likely to be affected by hemolysis. Several of the hemolysis-susceptible microRNAs (e.g., miRs-21, -106a, -92a, -17, -16) have also been previously proposed as plasma/serum biomarkers of disease, highlighting the importance of rigorous quality control of plasma/serum samples used for measurement of circulating microRNAs. As low-level hemolysis is a frequent occurrence during plasma/serum collection it is critical that this is taken into account in the measurement of any candidate circulating microRNA.

## Introduction

Since the discovery that microRNAs are not only present within cells, but can also be detected extracellularly in a variety of body fluids, a large number of studies have investigated the potential use of these cell-free microRNAs as diagnostic and/or prognostic biomarkers (Reid et al., 2011; Creemers et al., 2012; Mo et al., 2012). Unlike their longer mRNA counterparts which are prone to degradation in body fluids as a result of the high content of RNase particularly in blood, microRNAs are surprisingly stable. This increased stability of microRNAs can be attributed to: (i) their encapsulation into microvesicles such as exosomes and apoptotic bodies (Cortez and Calin, 2009; Kosaka et al., 2010) and (ii) their association with protein complexes such as argonaute 2 (Arroyo et al., 2011; Turchinovich et al., 2011) and high density lipoprotein (Vickers et al., 2011).

Being easily accessible and collected routinely as part of medical assessments, plasma and serum represent the most promising and best studied sources of cell-free microRNAs. While a large number of studies have aimed to identify microRNAs in plasma or serum that can serve as biomarkers for disease, there is still a lack of consensus regarding optimal quantification methods, appropriate reference genes as well as quality assurance and quality control of samples.

Of particular relevance to the identification of cell-free microRNA-based biomarkers in blood, a small number of studies have recently shown that rupturing of red blood cells (RBCs) occurring most often during blood collection or sample processing can have substantial impact on the levels of certain microRNAs detectable in plasma and serum (Kirschner et al., 2011; McDonald et al., 2011; Pritchard et al., 2012). These studies only investigated the effect of hemolysis on a very limited number of cell-free microRNAs, in particular miR-16 and miR-451, but serve as a first indication and warning that hemolysis can significantly alter the levels of microRNAs in plasma and serum. It is not surprising that the levels of miR-16 and miR-451 vary depending on the degree of hemolysis given they represent two of the most abundant microRNAs in RBCs (Bruchova et al., 2007; Vasilatou et al., 2010). However, our own data (Kirschner et al., 2011) and those of Pritchard et al. (2012) also revealed that levels of miR-92a, a microRNA proposed as a potential biomarker for ischemic heart disease (Fichtlscherer et al., 2010) and various cancer types (Tanaka et al., 2009; Huang et al., 2010; Ohyashiki et al., 2011) are also affected by hemolysis. In addition, Blondal et al. (2013) have recently reported on 119 microRNAs measured in high quality and compromised (general blood cell contamination) serum samples. This study showed that in samples with blood cell contamination many of those 119 microRNAs significantly deviate from the mean expression levels observed in 381 high quality samples used as comparator. Together, these observations raise the possibility that other potential biomarkers could be equally impacted by hemolysis.

In the present study we profiled the microRNA content of non-hemolyzed and hemolyzed plasma as well as RBCs to identify those microRNA most likely to be affected by hemolysis.

## Materials and Methods

### Blood collection

Blood was collected from consenting patients [with either malignant pleural mesothelioma (MPM) or coronary artery disease (CAD)] and healthy controls from the antecubital fossa using a butterfly device (21 G, BD Bioscience). A total of four 4 ml or three 10 ml K_3_EDTA Vacutainer Plus Tubes (BD Biosciences) was taken in one collection. Written informed consent was obtained from all participants and the study was approved by the Human Research Ethics Committee at Concord Repatriation General and Royal Prince Alfred Hospitals, Sydney, Australia. Within 60 min of blood collection samples were subjected to centrifugation at 2500 *g* for 20 min at room temperature. Plasma supernatant was removed leaving at least a 500 μl layer behind to avoid disturbing the buffy coat layer. Samples were frozen as 500 μl aliquots and stored at −80°C until further use. Purified RBCs from one healthy donor were obtained by separation of blood components using Ficoll-Paque PLUS according to the manufacturer’s recommendations. The dilution series used in this study was the same as published previously (Kirschner et al., 2011). Table 1 summarizes patient/volunteer demographics and identifies how samples were used in profiling and validation.

**Table 1:** **Patient/volunteer demographics and use of samples in this study**.

Sample ID	Gender	Age	Diagnosis	Profiling	Validation
N1*	F	31	N/A	No	Yes
N2*	F	31	N/A	Yes	Yes
MPM1	M	64	Sarcomatoid malignant mesothelioma	Yes	Yes
CAD1	M	79	Stable coronary artery disease	Yes	Yes
CAD2	M	65	Stable coronary artery disease	No	Yes
CAD3	M	80	Stable coronary artery disease	No	Yes

*N1 and N2 are the same individual with samples obtained from two independent collections (2 months apart) which both resulted in collection of 1 hemolyzed and 1 non-hemolyzed plasma sample with different degrees hemolysis. Blood for the dilution series was collected from the same individual.

We observed that blood collection of single patients sometimes resulted in plasma with hemolysis occurring in one or two of the collection tubes while the plasma in the remaining tube(s) was non-hemolyzed. The hemolyzed sample of the matched pairs used in this study was the result observed after standard collection procedure, and was not induced chemically or physically.

Hemolysis in the dilution series was achieved by addition of lysed RBCs (freeze-thawed and mixed by continuous vortexing for 60 s) to non-hemolyzed plasma from the healthy volunteer. Serial dilution from this 2% RBC sample was performed to obtain the samples used in this study, as described (Kirschner et al., 2011). Two independent dilution series using plasma from two independent blood collections were prepared.

### Assessment of hemolysis

The level of hemolysis in plasma samples was assessed by spectrophotometry (NanoPhotometer P300, Implen) with readings at wavelengths scanning from 350 to 650 nm. The degree of hemolysis was determined based on the optical density at 414 nm (absorbance peak of free hemoglobin), with additional peaks occurring at 541 and 576 nm being indicative of very high levels of hemolysis. Samples were classified as being hemolyzed if the A_414_ reading exceeded a valued of 0.2 as we have previously shown that excluding samples with higher A_414_ significantly decreases the variability of miR-16 and miR-451 (microRNAs highly abundant in RBCs) within a sample series (Kirschner et al., 2011). Based on our experience non-hemolyzed samples present with an A_414_ reading between 0.14 and 0.18. See Table 2 for A_414_ measurements of all samples used in this study.

**Table 2:** **Free hemoglobin measurements for matching sample pairs and dilution series**.

Sample	A_414_		Degree of hemolysis relative to non-hemolyzed
	Non-hemolyzed	Hemolyzed	
N1	0.174 ± 0.015	0.413 ± 0.062	2.36 ± 0.16
N2	0.153 ± 0.015	0.412 ± 0.039	2.68 ± 0.003
MPM1	0.124 ± 0.014	0.558 ± 0.01	4.53 ± 0.58
CAD1	0.184 ± 0.028	0.25 ± 0.025	1.37 ± 0.07
CAD2	0.127 ± 0.008	0.242 ± 0.012	1.90 ± 0.01
CAD3	0.143 ± 0.03	0.574 ± 0.129	4.01 ± 0.06
0.0% RBC	0.143 ± 0.013	N/A	N/A
0.008% RBC		0.184 ± 0.001	1.29 ± 0.13
0.016% RBC		0.245 ± 0.042	1.73 ± 0.45
0.031% RBC		0.321 ± 0.057	2.28 ± 0.61
0.0625% RBC		0.420 ± 0.036	2.96 ± 0.52
0.125% RBC		0.626 ± 0.019	4.39 ± 0.27

### RNA isolation

Total RNA was isolated from 400 μl of plasma from six individuals for whom non-hemolyzed and hemolyzed plasma obtained from different collection tubes but the same blood collection was available. In addition RNA was isolated from 100 μl Ficoll-purified RBCs from the healthy donor and from 400 μl of plasma containing RBC dilution [5 points of a serial dilution series (Kirschner et al., 2011)]. RNA isolation was performed using the mirVana PARIS miRNA isolation Kit (Life Technologies) with small modifications. The RBC samples were mixed with 300 μl cell disruption buffer prior to further processing identical to the plasma samples. Following the denaturing step of the isolation process, 100 μg mussel glycogen (Roche) were added to each sample as carrier to enhance isolation efficiency. After separation and recovery of the aqueous phase, a second phenol–chloroform extraction of the aqueous phase was included to improve the removal of the high protein content of plasma. Following the column-purification part, RNA was eluted using 100 μl ultrapure H_2_O (95°C), resulting in a recovery of around 85 μl RNA. For each sample two independent RNA isolations were performed.

For those samples to be used for microRNA profiling 70 μl of the total RNA were further concentrated in order to increase the amount of RNA that could be reverse transcribed. These samples were mixed with 300 mM sodium acetate pH 5.2, 2.5 ng mussel glycogen, and 175 μl 100% Ethanol and incubated over night at −80°C. RNA was precipitated by centrifugation at 17000 *g* for 20 min at 4°C. Pellets were washed with 1 ml 75% Ethanol and re-precipitated at 17000 *g* for 10 min at 4°C. After removal of the supernatant RNA was air-dried for approximately 10 min (until the RNA pellet changed color from white to opaque) and then resuspended in 10 μl ultrapure H_2_O. RNA concentration was assessed using the Qubit RNA Assay Kit (Life Technologies), however RNA concentrations for the plasma samples were below the limits of detection. All samples were stored at −80°C until further use.

### TaqMan array microfluidic card microRNA profiling

MicroRNA profiling was performed using the TaqMan Array Human MicroRNA A+B Cards Set v3.0 together with the Megaplex™RT Primers, Human Pool Set v3.0 (Life Technologies) following the protocol for profiling without pre-amplification.

The profiling was performed twice using two independent isolations of matching non-hemolyzed and hemolyzed plasma pairs from three individuals. Each RNA sample was reverse transcribed using each the A and B Megaplex Primer Pools. Briefly, 3 μl precipitated RNA was mixed with 1 reaction buffer, 3 mM MgCl_2_, 2 U RNase Inhibitor, 2.7 mM dNTPs, 1 Megaplex Primer Pool A or B, and 75 U MultiScribe Reverse Transcriptase under the following reaction conditions: 40 cycles of 2 min annealing at 16°C, complimentary DNA (cDNA) synthesis for 1 min at 42°C and 1 s at 50°C, followed by denaturing for 5 min at 85°C. The obtained cDNA was then stored at −20°C for use within 1 week.

Six microliters cDNA were mixed with 444 μl ultrapure H_2_O and 450 μl 2× TaqMan Gene Expression Master Mix and 100 μl of this mix were loaded into each port of the corresponding Microfluidic Array card according to the manufacturer’s instructions. Array cards were then run on a ViiA 7 Real-Time instrument (Life Technologies) with 2 min UDG incubation at 50°C and 10 min enzyme activation at 95°C followed by 40 cycles of 15 s denaturation at 95°C and 60 s annealing/elongation at 60°C.

Quantification cycle (Cq) values were determined using the ViiA 7 Software v1.2 applying a fixed threshold level of 0.05. Data were then analyzed using the 2^−ΔCq^ method (Livak and Schmittgen, 2001; Schmittgen and Livak, 2008) expressing levels of microRNAs in hemolyzed samples relative to those in non-hemolyzed samples without normalization to an endogenous control: relative expression = 2^−((Cqtarget(Hemolyzed)) − (Cqtarget(Non-Hemolyzed)))^. Alternatively, direct comparisons were performed at the level of raw Cq values. MicroRNAs with Cq value >35 in every sample analyzed were excluded from analysis.

### Individual microRNA reverse transcription real-time quantitative PCR

Levels of selected microRNAs were assessed using TaqMan microRNA Assays. Reverse transcription (RT) was performed using microRNA-specific stem-loop RT primers and the MicroRNA RT Kit (both Life Technologies). A fixed volume of 1.67 μl total RNA was used as template in the RT reaction, and combined with 4 μl of an equimolar mix of 7 or 8 microRNA-specific primers (consisting of 31.25 nM of each specific primer), 1 nM dNTPs, 2.4 U RNase Inhibitor, 33 U MultiScribe reverse transcriptase, and RNase-free water in a total volume of 10 μl. RT primer mix 1 consisted of primers for miR-122 (ID 002245), -142-3p (ID 000464), -146a (ID 000468), -16 (ID 000391), -486-3p (ID 002093), -532-3p (ID 002355), -636 (ID 002088), and -886-5p (ID 002193), mix 2 contained primers for miR-1255B (ID 002801), -1274B (ID 002884), -15b (ID000390), -16, -451 (ID 001105), -625-3p (ID 002432), and RNU48 (001006), mix 3 consisted of miR-103 (ID 000439), -106a (ID 002169), -126 (ID 002228), -17 (ID 000393), -27a (ID 000408), -29a (ID 002112), and -92a (ID 000431), and mix 4 contained miR-155 (ID 002623), -16, -21 (ID 000397), 210 (ID 000512), -223 (002295), -31 (ID 002279), and -720 (ID 002895). Reaction conditions followed the manufacturer’s instructions: annealing for 30 min at 16°C, followed by cDNA synthesis for 30 min at 42°C and denaturing for 5 min at 85°C. The resultant cDNA was diluted by addition of 57.8 μl water and used immediately in qPCR or stored at −20°C.

For real-time qPCR detection of microRNAs, 2.25 μl of the diluted cDNA were used as template in reactions containing 1× microRNA-specific TaqMan primers/probes (see above for assay IDs) in combination with 1× TaqMan GeneExpression MasterMix (both Life Technologies) according to the manufacturer’s instruction in a total reaction volume of 10 μl, with the following reaction conditions: 2 min UDG incubation at 50°C and 10 min enzyme activation at 95°C followed by 40 cycles of 15 s denaturation at 95°C and 60 s annealing/elongation at 60°C. No-template samples were included as negative controls. Duplicate qPCR reactions were set up manually and run on a ViiA 7 Real-Time Instrument (Life Technologies). Cq values were determined using the ViiA 7 Software v1.2 applying a fixed threshold level of 0.05. In case of matched pairs of non-hemolyzed and hemolyzed plasma, relative abundance in the hemolyzed samples as compared to the non-hemolyzed sample was calculated using the 2^−ΔCq^ method (Livak and Schmittgen, 2001; Schmittgen and Livak, 2008) as described above. Data for the dilution series are presented as raw Cq values for each microRNA.

All raw Cq data (average of duplicates) are provided in Tables 1 (profiling) and 2 (validation) in Supplementary Material. Minimum Information for Publication of Quantitative Real-Time PCR Experiments (MIQE) guidelines (Bustin et al., 2009) were followed for description of samples, RNA extraction, RT, qPCR protocol, and data analysis.

## Results

### Profiling matched hemolyzed and non-hemolyzed plasma samples identifies microRNAs affected by hemolysis

In order to better understand the contribution of hemolysis to the levels of microRNAs in plasma, we profiled the microRNA content of three matched pairs of non-hemolyzed and hemolyzed plasma and one RBC sample from the healthy donor.

**Figure 1:**
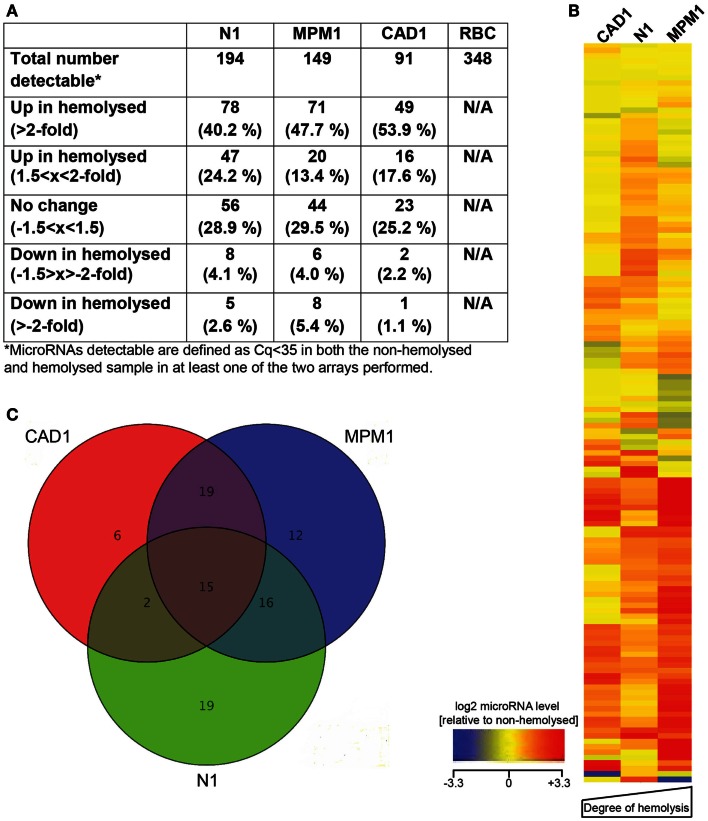
**MicroRNA profiling of hemolyzed and non-hemolyzed plasma**. **(A)** Summary of each pairs microRNA profile, representing the total number of microRNAs detectable as well as numbers of microRNAs affected or unaffected by hemolysis in each pair. **(B)** Heatmap of relative abundance of 136 microRNAs in hemolyzed compared to non-hemolyzed plasma. Levels of microRNAs in hemolyzed samples are presented as log2 of the relative expression level (non-hemolyzed = 0), with ±3.3 being the equivalent of a ±10-fold difference in relative abundance. The microRNAs presented are those detectable in at least four out of the six plasma samples investigated. **(C)** Overlap of microRNAs increased >2-fold in each of the three pairs. Fifteen microRNAs (inner triangle) were >2-fold increased in all three investigated pairs, and an additional 37 microRNAs were >2-fold increased in at least two of the investigated pairs.

The number of microRNAs detected with a Cq < 35 in each sample varied between 91 and 194, as did the number of microRNAs affected by hemolysis (Figure 1A). We further filtered these microRNAs, identifying a total of 136 microRNAs that were detectable in at least 4 of the 6 plasma samples profiled and, with the exception of the liver-specific miR-122, all of these were also detected in the RBC sample. Relative expression levels of those 136 microRNAs in the hemolyzed sample as compared to the non-hemolyzed counterpart are pictured in Figure 1B. This shows that the majority of detectable microRNAs are elevated in hemolyzed samples (represented by red color), while only a handful of microRNAs are present at lower levels in hemolyzed plasma (blue). The heat map further shows that there is a general trend toward a correlation of higher levels of hemolysis-susceptible microRNAs with the degree of hemolysis.

**Figure 2:**
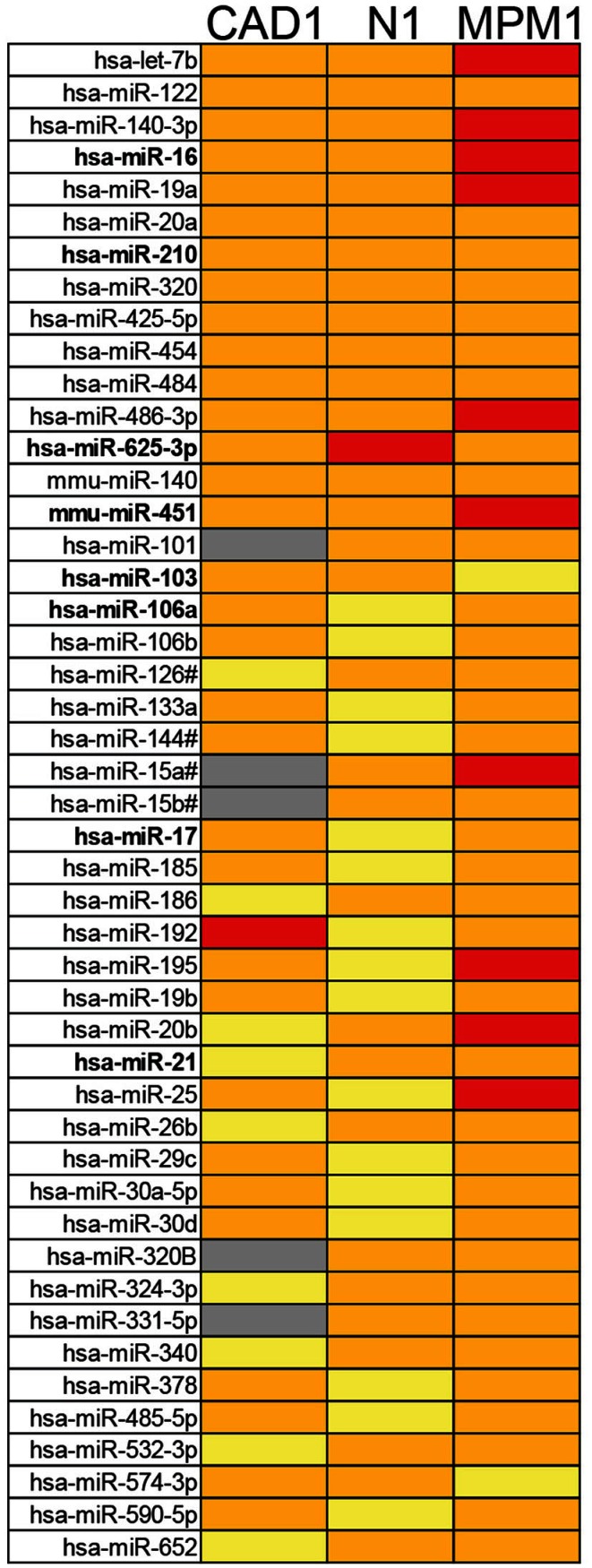

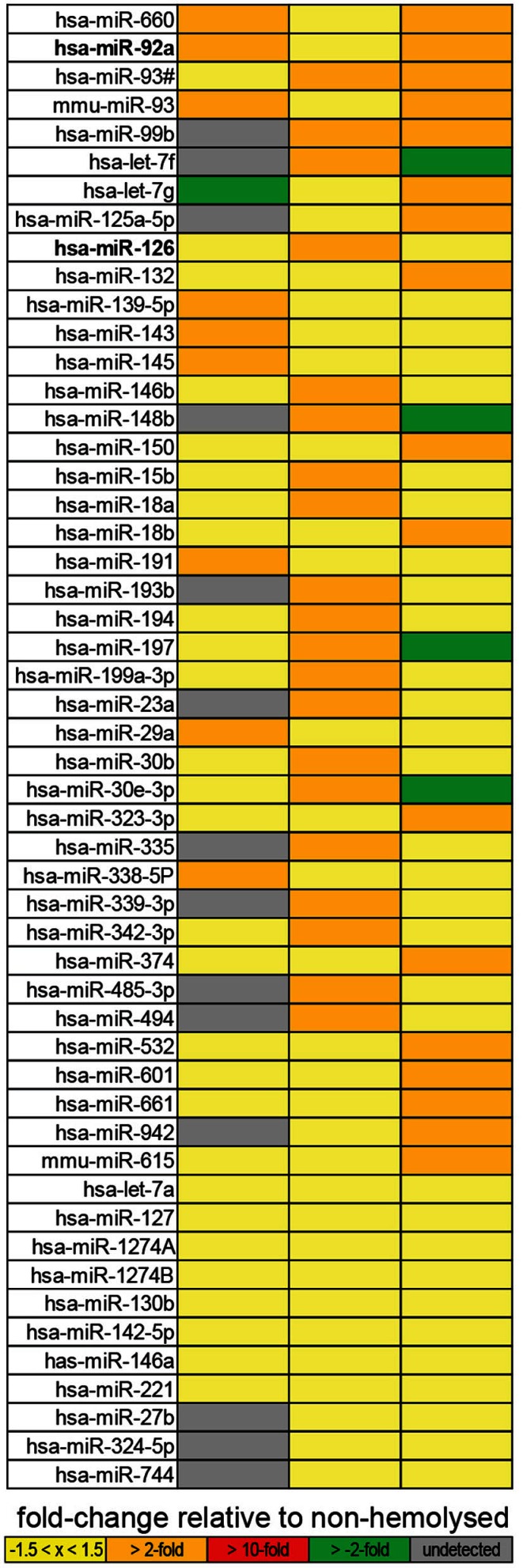
**Overview of microRNAs identified as being elevated in hemolyzed plasma or unaffected by hemolysis**. The top 15 microRNAs are elevated in the hemolyzed sample of all three pairs, the following 37 microRNAs are elevated in two of the three pairs followed by the 36 microRNAs elevated in just one pair. The 11 microRNAs at the bottom of the list represent those with less than ±1.5-fold difference between the hemolyzed and non-hemolyzed sample.

Further analysis identified that of the microRNAs detected in plasma, 15 (11%, the center of the Venn diagram in Figure 1C) demonstrated >2-fold higher levels in the hemolyzed samples of all three pairs investigated. The number of elevated microRNAs in hemolyzed compared to non-hemolyzed plasma increased to 52 (38.2%) microRNAs when including those only elevated in two of the three pairs and to a total of 88 (64.7%) microRNAs being elevated >2-fold in at least one of the pairs. Those microRNAs identified as being significantly altered in only one of the pairs were generally also altered in the other pairs, but did not show increases above the selected threshold of at least twofold. Of the microRNAs detectable only 11 appeared to be truly unaffected by hemolysis and did not vary by more than ±1.5-fold in any of the investigated pairs. Our profiling studies also identified 3 microRNAs (miR-1255B, miR-636, miR-886-5p) and a small nucleolar RNA (snoRNA, RNU48) which were only present in RBCs and hemolyzed plasma. While additional microRNAs were found to be present only in RBCs and one or two of the hemolyzed samples, these were not subject to further validation due to Cqs being >35. Figure 2 provides an overview of all microRNAs which increased in relative abundance by more than twofold following hemolysis as well as those unaffected by hemolysis.

### Changes of selected microRNAs with increasing RBC contamination

Our profiling data suggested that the levels of a number of microRNAs detected in plasma increased with hemolysis. To further investigate the contribution of RBC microRNAs to plasma levels, we measured the levels of selected microRNAs in a series of 5 RNA samples obtained from a serial dilution of RBCs in non-hemolyzed plasma (Kirschner et al., 2011) and six matched pairs of non-hemolyzed and hemolyzed plasma (the three pairs used for profiling +3 more). We quantified two microRNAs affected by hemolysis (miR-486-3p, miR-532-3p), three not affected by hemolysis (miR-1274B, miR-142-3p, miR-146a), and the four microRNAs/snoRNAs only present in RBCs and hemolyzed plasma (miR-1255B, miR-636, miR-886-5p, RNU48). In addition we included miR-16, miR-15b, and miR-451 as miRs previously shown to be affected by hemolysis as well as the liver-specific miR-122.

**Figure 3:**
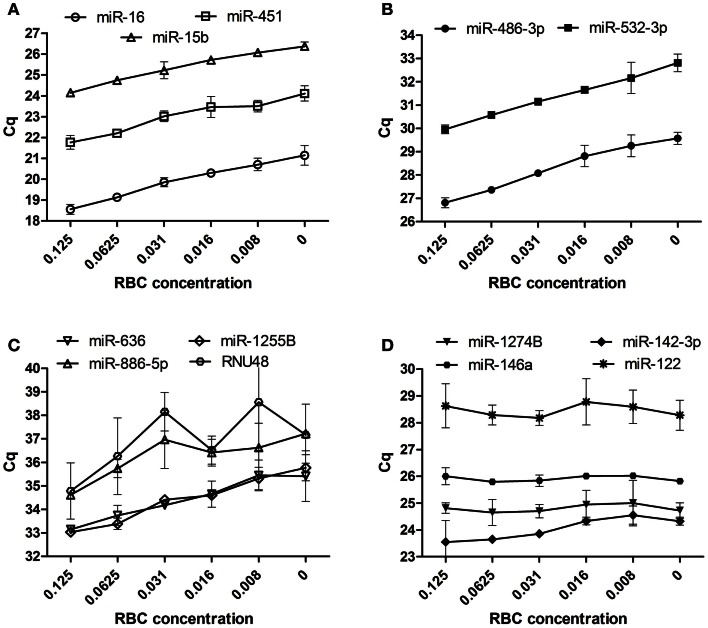
**RT-qPCR validation of candidates in dilution series of RBCs in plasma**. **(A)** Previously identified hemolysis affected microRNAs **(B)** additional hemolysis affected microRNAs **(C)** RBC- and hemolyzed plasma specific microRNAs **(D)** unaffected microRNAs. Data are presented as raw Cq value ± SD obtained from measurements in RNA isolated from two independent dilution series.

Using our RBC dilution series, we observed an increase in Cq values for miR-16, miR-451, and miR-15b with increasing RBC concentration (Figure 3A), consistent with previously published data (Kirschner et al., 2011; McDonald et al., 2011). In addition, we observed a considerable decrease in Cq (and therefore an increase in abundance) with increasing RBC contamination for the hemolysis-susceptible microRNAs miR-486-3p and miR-532-3p identified from the profiling (Figure 3B). Similarly, levels of miRs-1255B, -636, -886-5p, and RNU48, microRNAs/snoRNAs identified as being RBC- and hemolysis-specific, also increased with increasing RBC content (Figure 3C). In contrast levels of those microRNAs identified as being the most stable (miR-142-3p, miR-146a, and miR-1274B), as well as the liver-specific miR-122 did not change with increasing RBC concentration and showed only minor variability with <1.1 cycles between the highest and lowest Cq for any of those miRs (Figure 3D;Table 3).

**Table 3:** **Cq values of stable miRs in dilution series**.

RBC concen-tration (%)	miR-1274B	miR-142-3p	miR-146a	miR-122
0.0	24.73 ± 0.29	24.33 ± 0.15	25.82 ± 0.06	28.28 ± 0.56
0.008	25.00 ± 0.85	24.55 ± 0.34	26.02 ± 0.08	28.59 ± 0.62
0.016	24.94 ± 0.54	24.34 ± 0.14	26.01 ± 0.08	28.78 ± 0.86
0.031	24.70 ± 0.24	23.86 ± 0.12	25.84 ± 0.21	28.18 ± 0.28
0.0625	24.65 ± 0.49	23.65 ± 0.08	25.80 ± 0.05	28.29 ± 0.37
0.125	24.82 ± 0.20	23.55 ± 0.80	26.00 ± 0.31	28.63 ± 0.82

**Figure 4:**
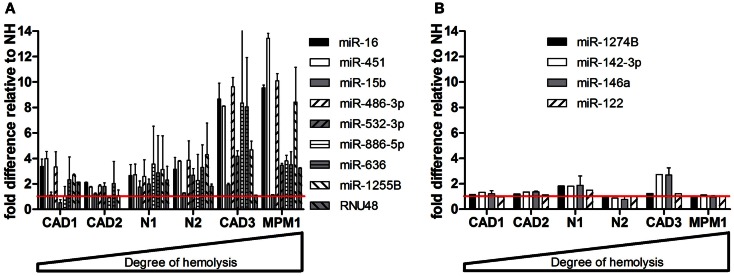
**Validation of microRNAs in matching pairs**. **(A)** microRNAs changing with hemolysis **(B)** microRNAs not affected by hemolysis. Data are presented as relative microRNA level ± SD in the hemolyzed compared to the non-hemolyzed sample value obtained from measurements in RNA from two independent isolations per sample. The red line represents the relative expression of the corresponding non-hemolyzed samples.

When measuring levels of these microRNAs in matched pairs of non-hemolyzed and hemolyzed plasma, we found a similar pattern to that observed in the dilution series. The microRNAs shown to be influenced by RBC content of the sample – miR-16, -451, -15b, 486-3p, 532-3p, -886-5p, 636, -1255B, and RNU48 – were up to 13-times more abundant in the hemolyzed sample as compared to its non-hemolyzed counterpart (Figure 4A). In addition this increase in abundance was correlated with the degree of hemolysis observed for the corresponding sample. MicroRNAs identified as being unaffected by hemolysis showed only minor variation (<±1.5-fold difference) in relative expression between hemolyzed and non-hemolyzed plasma (with the exception of CAD3) (Figure 4B).

### Impact of hemolysis on proposed biomarker candidates

**Figure 5:**
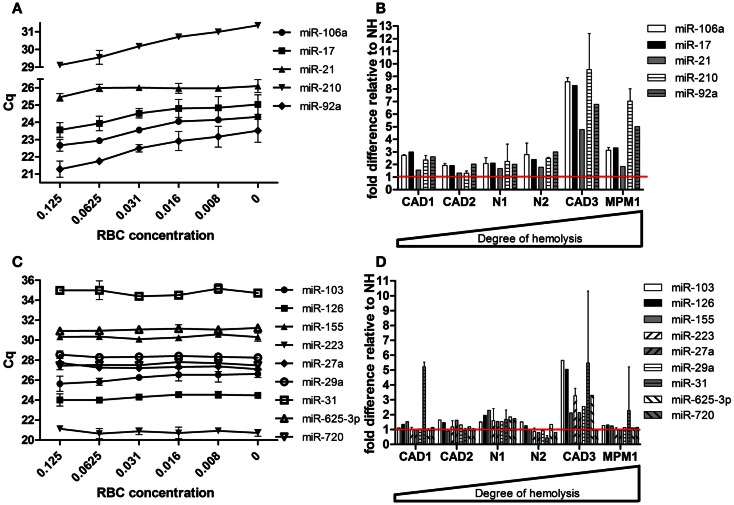
**Effect of hemolysis on biomarker candidates**. Five proposed biomarker candidates were identified to be changing with increased hemolysis in **(A)** the RBC dilution series and **(B)** the six matching pairs of hemolyzed and non-hemolyzed plasma. Another nine proposed biomarkers remained unaffected by increasing hemolysis in both the dilution series **(C)** and the matched pairs **(D)**. The red line represents the relative expression of the corresponding non-hemolyzed samples. Data are presented as raw Cq value ± SD **(A,C)** or relative microRNA level ± SD **(B,D)**.

Following validation of the microRNAs selected from our profiling studies, we investigated the effect of hemolysis on microRNAs previously proposed as biomarkers for disease (Table 4). These microRNAs included those for which the profiling suggested an influence of hemolysis (miR-103, -106a, 17, -21, -210, -27a, -31, -625-3p, -92a) as well as those unaffected by RBC contamination (miR-122, -126, -146a, -155, -223, -29a, -720). Measurement in the dilution series confirmed that microRNAs miR-106a, miR-17, miR-92a, and miR-210 are significantly affected by the level of RBC contamination of the sample displaying decreases in Cq values of 1.5–2.4 cycles between 0 and 0.125% RBC contamination (Figure 5A). This was also confirmed when looking at the matched pairs in which those miRs were in most cases at least twofold higher in the hemolyzed sample (Figure 5B). In addition miR-21 seemed to be affected by high degrees of hemolysis (Figure 5B). Although miR-31 levels appear to be higher in hemolyzed compared to non-hemolyzed plasma, this variability could be a result of the low expression of this microRNA, with Cq values being >34 in all samples. None of the remaining microRNAs showed a correlation between Cq values and the degree of hemolysis (Figures 5C,D).

**Table 4:** **Proposed biomarker microRNAs**.

miRNA	Disease	Regulation	Reference	Affected by hemolysis
miR-103	MPM	Down (compared to asbestos-exposed and healthy)	Weber et al. (2012)	No
	Pancreatic cancer (PC)	Up (compared to healthy)	Ren et al. (2012)	
miR-106a	Gastric cancer (GC)	Up (compared to healthy)	Tsujiura et al. (2010)	Yes
miR-122	Hepatitis B virus (HBV)-related hepatocellular carcinoma (HCC)	Up (compared to healthy)	Zhou et al. (2011)	No
	Hepatitis B	Up (compared to healthy)	Zhang et al. (2010)	
	Liver injury (mouse model)	Up (compared to no injury)	Zhang et al. (2010)	
miR-126	MPM	Down (compared to healthy and NSCLC)	Santarelli et al. (2011), Tomasetti et al. (2012)	No
	Abdominal aortic aneurysm (AAA)	Down (compared to healthy)	Kin et al. (2012)	
miR-146a	Esophagitis	Up (compared to healthy)	Lu et al. (2012a)	No
	PC	Down (compared to healthy)	Ali et al. (2010)	
miR-155	AAA	Down (compared to healthy)	Kin et al. (2012)	No
	Esophageal cancer	Down (compared to healthy)	Liu et al. (2012)	
	Breast cancer	Up (compared to healthy)	Lu et al. (2012b)	
miR-16	Traumatic brain injury	Up (compared to healthy)	Redell et al. (2010)	Yes
	Myelodysplastic syndrome	Up (compared to healthy)	Zuo et al. (2011)	
miR-17	Endometriosis	Down (compared to healthy)	Jia et al. (2013)	Yes
	GC	Up (in later stages)	Wang et al. (2012)	
	GC	Up (compared to healthy)	Tsujiura et al. (2010)	
miR-21	PC	Up (compared to healthy)	Ali et al. (2010)	Yes
	GC	Up (compared to healthy)	Tsujiura et al. (2010)	
	HBV-related HCC	Up (compared to healthy)	Zhou et al. (2011)	
	Esophagitis	Up (compared to healthy)	Lu et al. (2012a)	
miR-210	PC	Up (compared to healthy)	Wang et al. (2009)	Yes
	Kidney injury	Up (compared to healthy)	Lorenzen et al. (2011)	
miR-223	NSCLC	Down (in later stages)	Heegaard et al. (2012)	No
	AAA	Down (compared to healthy)	Kin et al. (2012)	
	Esophagitis	Up (compared to healthy)	Lu et al. (2012a)	
	HBV-related HCC	Down (compared to healthy)	Zhou et al. (2011)	
miR-27a	HBV-related HCC	Down (compared to healthy)	Zhou et al. (2011)	No
miR-29a	CRC	Up (compared to healthy)	Huang et al. (2010)	No
miR-31	Breast cancer	Up (compared to healthy)	Lu et al. (2012b)	No
miR-451	GC	Up (compared to healthy)	Konishi et al. (2012)	Yes
miR-625-3p	MPM	Up (compared to healthy and asbestosis)	Kirschner et al. (2012)	No
miR-720	Multiple myeloma	Up (compared to healthy)	Jones et al. (2012)	No
miR-92a	CAD	Down (compared to healthy)	Fichtlscherer et al. (2010)	Yes
	Non-Hodgkin’s lymphoma	Down (compared to healthy)	Ohyashiki et al. (2011)	
	Multiple myeloma	Down (compared to healthy)	Yoshizawa et al. (2012)	
	CRC	Up (compared to healthy)	Huang et al. (2010)	

### Identification of microRNA subsets displaying similar changes due to hemolysis

The clear influence of hemolysis on plasma microRNA content raises the question of whether hemolyzed samples can be included in biomarker discovery studies. To further address this issue we interrogated the profiling data in an attempt to identify groups of microRNAs with similar patterns of hemolysis-induced increase with the aim of identifying subsets of microRNA from which both endogenous controls and potential biomarkers could be derived. For the purpose of this part of the study we focused on the two samples exhibiting extensive hemolysis. Twenty-six microRNAs were increased by at least twofold, in these samples with increases following the degree of hemolysis (Table 5). Similarly, we identified a second subset of 19 microRNAs which varied by <±1.5-fold (Table 5). Both groups include microRNAs previously proposed as biomarkers for disease. Although requiring further validation, these two subsets represent groups of microRNAs that may be used for selection of biomarker candidate(s) and endogenous control(s) potentially enabling hemolyzed samples to be included in biomarker discovery studies.

**Table 5:** **Subsets of biomarker-endogenous control candidates**.

microRNAs changing with hemolysis			microRNAs unaffected by hemolysis		
microRNA	Relative fold increase H vs. NH		microRNA	Relative fold difference H vs. NH	
	N1	MPM1		N1	MPM1
let-7b	2.12 ± 0.43	14.06 ± 9.83	let-7a	1.33 ± 1.3	1.25 ± 0.71
miR-126#	2.70 ± 1.03	2.87 ± 1.00	let-7d	1.49 ± 0.77	0.97 ± 0.78
miR-140-3p	2.60 ± 0.70	10.96 ± 3.69	miR-1260	1.37 ± 1.14	1.23 ± 0.52
miR-15a#	2.92 ± 1.96	2.00 ± 1.10	miR-127	1.29 ± 0.68	0.66 ± 0.86
miR-15b#	5.40 ± 4.17	4.96 ± 2.04	miR-1274A	1.14 ± 0.27	1.20 ± 0.59
miR-16	3.55 ± 0.87	10.18 ± 0.38	miR-1274B	1.49 ± 0.39	0.87 ± 0.06
miR-193b	5.26 ± 3.39	6.16 ± 6.00	miR-130b	1.00 ± 0.38	1.10 ± 0.33
miR-194	18.14 ± 14.34	2.86 ± 2.21	miR-142-5p	1.50 ± 1.04	1.30 ± 0.43
miR-20a	2.10 ± 0.83	3.83 ± 0.37	miR-143	0.89 ± 0.47	1.09 ± 0.32
miR-20b	2.46 ± 1.80	19.24 ± 3.30	miR-146a	1.29 ± 0.14	1.49 ± 0.87
miR-21	2.57 ± 0.47	2.90 ± 0.22	miR-221	1.35 ± 0.58	1.50 ± 0.93
miR-210	2.50 ± 1.01	26.93 ± 7.48	miR-222	1.26 ± 0.45	1.38 ± 0.06
miR-26b	2.56 ± 0.48	3.58 ± 1.29	miR-27b	1.49 ± 1.40	1.27 ± 0.21
miR-320	2.25 ± 0.13	3.32 ± 0.01	miR-324-5p	1.20 ± 0.04	1.08 ± 0.90
miR-320B	2.28 ± 0.96	3.66 ± 3.50	miR-338-5P	0.76 ± 0.06	1.41 ± 0.77
miR-324-3p	3.30 ± 1.51	9.12 ± 6.61	miR-339-5p	1.29 ± 0.94	1.04 ± 0.16
miR-331-5p	3.54 ± 3.40	22.65 ± 19.81	miR-340#	0.94 ± 0.38	0.8 ± 0.53
miR-340	2.63 ± 1.43	3.71 ± 1.20	miR-425#	1.10 ± 0.83	0.96 ± 0.02
miR-425-5p	2.44 ± 0.90	4.75 ± 3.74	miR-744	0.71 ± 0.21	1.01 ± 0.31
miR-454	4.62 ± 1.19	6.15 ± 3.64			
miR-484	2.11 ± 0.12	3.13 ± 0.09			
miR-486-3p	2.51 ± 1.32	14.20 ± 5.57			
miR-532-3p	2.50 ± 2.05	3.75 ± 2.71			
miR-652	3.08 ± 2.52	3.45 ± 0.43			
miR-140	2.17 ± 0.18	3.20 ± 0.56			
miR-451	2.38 ± 0.84	15.27 ± 1.78			

## Discussion

Cell-free microRNAs are detectable in a variety of body fluids and have in recent years attracted a lot of attention due to their potential use as biomarkers for disease. The majority of studies have focused on plasma or serum, attractive because it is readily available, can be collected with minimal risk or discomfort and is routinely collected as part of clinical assessment of patients. As a result a large number of novel plasma or serum microRNA biomarkers have been suggested in the last 5 years (Brase et al., 2010; Reid et al., 2011; Creemers et al., 2012; Mo et al., 2012). Nevertheless, despite this rapid growth the field still suffers from a lack of standardized and detailed reporting methods.

The cellular origin of most microRNAs detectable in the circulation is as yet unknown. Studies comparing the microRNA profile of plasma or serum from healthy and diseased individuals (Chen et al., 2008; Kirschner et al., 2012) have shown that there is extensive overlap between the profiles suggesting that many of the microRNAs present in the circulation could play important roles in the normal functioning of the circulatory and immune system. Consequently, proposed markers for disease rarely seem to be tissue-specific, making their use as markers for specific conditions difficult. Examples of such microRNAs are miR-92a [proposed as a diagnostic marker for colorectal cancer (CRC) (Huang et al., 2010), and Non-Hodgkin’s Lymphoma (Ohyashiki et al., 2011), as well as CAD (Fichtlscherer et al., 2010)] and miR-21 [e.g., in gastric cancer (Tsujiura et al., 2010; Li et al., 2012), CRC (Kanaan et al., 2012) and Non-small cell lung cancer (NSCLC) (Tang et al., 2013)]. Assessment of the true role of these microRNAs is further complicated by the high abundance of these and other microRNAs in blood cells, and by variations in blood cell counts that have been shown to have a significant impact on the levels detectable in plasma or serum (Duttagupta et al., 2011; Pritchard et al., 2012).

The influence of blood cell lysis on plasma and serum levels of microRNAs has until recently been neglected. While it had already been reported that lymphocytes and RBCs have specific microRNA content (Ramkissoon et al., 2006; Bruchova et al., 2007; Collino et al., 2010; Vasilatou et al., 2010), the overlap between these profiles and those in plasma were initially overlooked. In 2011, after 3 years of biomarker discovery studies, the first reports describing the effects of hemolysis occurring during blood collection or processing appeared, and suggested that this can have considerable impact on the levels of certain microRNAs detected in hemolyzed samples (Kirschner et al., 2011; McDonald et al., 2011; Pritchard et al., 2012). The three studies all identified miR-16 and miR-451 as being the most highly abundant microRNAs in RBCs, and found levels of these microRNAs in plasma to be most affected by hemolysis. In addition to the contribution of RBCs to the cell-free microRNA profile, Pritchard et al., also showed that platelet-derived microRNAs can significantly vary between samples. Most studies highlighting the problems associated with hemolysis have not yet gone beyond investigating small numbers of microRNAs (in particular miR-16 and miR-451), but two reports have already identified that miR-92a (a putative biomarker in several cancers) is heavily affected by hemolysis (Kirschner et al., 2011; Pritchard et al., 2012). In addition a recent study by Blondal et al. (2013) has compared levels of 119 microRNAs in high quality serum/plasma and compromised serum/plasma. This study has shown that the levels of many of those microRNAs deviate significantly from the average level observed in a large number of high quality samples when being measured in a compromised sample with high blood cell contamination. Furthermore, this study suggested the use of a ratio between a microRNA known to be highly variable with hemolysis (miR-451) and a microRNA found to be unaffected by hemolysis (miR-23a) as an indicator for hemolysis, especially in a situation where only RNA is available while the original plasma/serum sample is unavailable for assessment of hemolysis (Blondal et al., 2013).

To build on these observations and to further investigate the effect hemolysis has on proposed cell-free microRNA biomarker candidates we performed a more comprehensive comparison of the microRNA content in hemolyzed and non-hemolyzed plasma. This revealed up to 65% (88 of 136) of the microRNAs detectable in plasma to be elevated in hemolyzed samples (Figure 2). Among those were many microRNAs that have been previously proposed as biomarkers for various diseases (Table 4), and upon validation we demonstrate that in addition to miR-16, -451, and 92a, levels of miRs-106a, -17, -21, and -210 were susceptible to hemolysis (Figures 4A,B). This observation raises the question of whether microRNAs present in RBCs are suitable for use as biomarkers, even if they are often expressed at high levels in certain tissues or overexpressed in solid tumors. The enrichment of these microRNAs in RBCs complicates the interpretation of biomarker studies as it is difficult to discriminate between presence of a microRNA in the circulation due to controlled release from the tissue of origin (e.g., microvesicles), mechanical rupture of cells (e.g., ischemia) and the changes introduced by hemolysis.

Hemolysis may occur *in vivo* as part of the underlying disease process, or (more commonly) as a complication of the collection and processing of blood. Hematological malignancies such as chronic lymphocytic leukemia can result in autoimmune hemolysis (Rytting et al., 1996), while metastatic solid cancers can be associated with microangiopathic hemolysis and disseminated intravascular coagulation (Lohrmann et al., 1973; Rytting et al., 1996; Lechner and Obermeier, 2012). In addition, a number of drugs – in particular chemotherapeutic drugs – can influence the normal structure of RBCs (Dumez et al., 2004; Schauf et al., 2004), and render the cell vulnerable to damage as it passes through the microvasculature. This in turn can result in release of RBC intracellular contents, and therefore has the potential to increase RBC-enriched microRNAs in the plasma. However, these are all relatively rare events which in most patients would not be expected to contribute to a significant increase in RBC-related microRNAs. Besides the possibility of disease- or drug-associated hemolysis resulting in an increase in levels of RBC-enriched microRNAs, disease-related anemia has the potential to result in changes in the levels of potential biomarker microRNAs in plasma/serum. A number of studies have reported a decrease in levels of candidate microRNA biomarkers in cancer, which is somewhat counterintuitive. For example, reduced levels of the RBC-enriched miR-92a were reported to have diagnostic potential in hematological malignancies, while levels of this microRNA are increased in the corresponding malignant cells (Tanaka et al., 2009; Ohyashiki et al., 2011; Yoshizawa et al., 2012). In addition miR-126, another microRNA present at high levels in RBCs was found to be reduced in plasma from MPM patients (Santarelli et al., 2011; Tomasetti et al., 2012), a disease in which anemia is a relatively frequent occurrence. It is tempting to speculate that this somewhat surprising discrepancy between plasma and cell levels of miR-92a in leukemia, and the decrease of miR-126 in MPM, results from reduced levels of RBCs as a result of cancer-related anemia in those patients.

More commonly hemolysis occurs during blood collection and sample processing. Hemolysis has been reported in up to 42.6% of general medical hospital inpatients (free hemoglobin >0.5 g/l), but only 5.6% when using visual inspection (Lippi et al., 2009; Hawkins, 2010). Causes of sample hemolysis include venous stasis, phlebotomist experience and site of collection (Lippi et al., 2005; Hawkins, 2010). Using spectrophotometry we have detected significant hemolysis in approximately 15% of collected samples (often in the absence of color change in plasma/serum visible to the eye). Hemolysis may be an under-reported and important contributor to changes in microRNA levels in plasma or serum. The relative importance of a technical cause of hemolysis should be considered in the context of two different scenarios: biomarker discovery and diagnostic testing. In the case of studies aiming to identify microRNAs as diagnostic or prognostic markers for a disease, the effect of hemolysis may be limited as one would expect that hemolysis occurring during blood taking or plasma processing would affect equal numbers of samples in both case and control groups. Thus, variations introduced by *in vitro* hemolysis should therefore be present in any sample series investigated and not influence the identification of potential biomarkers. However, the large variations in abundance of microRNA biomarkers reported in a number of studies might be a result of the inclusion of hemolyzed samples. This speculation is supported by our own experience; when excluding hemolyzed plasma from our sample series we find a significant reduction in variability in levels of RBC-microRNA (Kirschner et al., 2011) and also see a lower variability in the levels of potential diagnostic markers (Kirschner et al., 2012) than those reported by others (Ng et al., 2009; Heneghan et al., 2010; Huang et al., 2010; Devaux et al., 2012). Together, these observations suggest that a quantification of hemolysis in samples should be included in any study of a potential biomarker candidate.

Although hemolysis is likely to represent only a minor confounding factor in biomarker discovery studies, the situation is different once a microRNA is actually confirmed as a marker for a specific disease and used as a diagnostic/prognostic tool. In this setting, where for instance a single sample is analyzed to determine presence or absence of disease, hemolysis can significantly impact on the result, especially if the microRNA used as marker is hemolysis-susceptible. As the clinical application of microRNAs as diagnostic/prognostic markers is the ultimate aim of the discovery studies currently performed, it is essential that these potential confounders are taken into consideration, and hemolysis needs to be quantified as part of the quality control process. While appropriate methods for normalization of microRNA expression data [generally obtained through real-time quantitative PCR (RT-qPCR)] could potentially overcome the problems associated with hemolysis-related variability, the normalization of plasma/serum microRNA data has yet to be standardized. The problem of normalization has been addressed in many studies as it is often not possible to accurately quantify RNA concentration, with the result that using equal amounts of RNA as input into the reactions is mostly impossible (Brase et al., 2010; Reid et al., 2011; Zampetaki and Mayr, 2012). A frequently used alternative is to spike-in known amounts of exogenous microRNAs (such as *C. elegans* miRs) during the isolation process (Mitchell et al., 2008). This approach certainly has the potential to account for differences in RNA isolation efficiency between different samples, but it is not able to account for differences introduced by hemolysis. Based on the profiling data in the present study, we propose the possibility of selecting a biomarker and an appropriate endogenous control from subsets of microRNAs affected equally by hemolysis. Our pilot study identified two potential subsets, one with microRNAs unaffected by hemolysis and one with hemolysis-susceptible microRNAs. Such an approach can serve as a starting point for further in-depth validation in a much larger sample set.

In summary, our data provide further evidence that hemolysis has a substantial impact on a large number of cell-free microRNAs in plasma and serum, many of which are being investigated as potential biomarkers of disease. These data suggest that future investigations of cell-free microRNA biomarkers should carefully assess hemolysis and the effect it has on a biomarker candidate.

## Conflict of Interest Statement

The authors declare that the research was conducted in the absence of any commercial or financial relationships that could be construed as a potential conflict of interest.

## Supplementary Material

The Supplementary Material for this article can be found online at http://www.frontiersin.org/Non-Coding_RNA/10.3389/fgene.2013.00094/abstract
